# Assessing the structure and diversity of fungal community in plant soil under different climatic and vegetation conditions

**DOI:** 10.3389/fmicb.2023.1288066

**Published:** 2023-11-29

**Authors:** Sen Liu, Chengjie Xiong, Longbing Lin, Nemat O. Keyhani, Mengjia Zhu, Zhiying Zhao, Weibin Zhang, Chenjie Yang, Hailan Su, Pu Liu, Xiayu Guan, Junzhi Qiu

**Affiliations:** ^1^Key Lab of Biopesticide and Chemical Biology, Ministry of Education, State Key Laboratory of Ecological Pest Control for Fujian and Taiwan Crops, College of Life Sciences, Fujian Agriculture and Forestry University, Fuzhou, Fujian, China; ^2^Department of Biological Sciences, University of Illinois, Chicago, IL, United States; ^3^Crop Institute of Fujian Academy of Agricultural Sciences, Fuzhou, China; ^4^Engineering Research Center of Edible and Medicinal Fungi, Ministry of Education, Jilin Agricultural University, Changchun, China; ^5^College of Horticulture, Fujian Agriculture and Forestry University, Fuzhou, Fujian, China

**Keywords:** ecological diversity, fungal communities, geological and environmental factors, functional and structural traits, fungal diversity

## Abstract

**Introduction:**

Understanding microbial communities in diverse ecosystems is crucial for unraveling the intricate relationships among microorganisms, their environment, and ecosystem processes. In this study, we investigated differences in the fungal community structure and diversity in soils from two contrasting climatic and vegetation conditions: the Xinjiang western China plateau and the Fujian southeastern coastal province.

**Methods:**

A total of 36 soil samples collected from two climatic regions were subjected to high-throughput ITS gene sequencing for fungal community analysis. In conjunction soil physicochemical properties were assessed and compared. Analyses included an examination of the relationship of fungal community structure to environmental factors and functional profiling of the community structure was using the FUNGuild pipeline.

**Results:**

Our data revealed rich fungal diversity, with a total of 11 fungal phyla, 31 classes, 86 orders, 200 families, 388 genera, and 515 species identified in the soil samples. Distinct variations in the physicochemical properties of the soil and fungal community structure were seen in relation to climate and surface vegetation. Notably, despite a colder climate, the rhizosphere soil of Xinjiang exhibited higher fungal (α-)diversity compared to the rhizosphere soil of Fujian. β-diversity analyses indicated that soil heterogeneity and differences in fungal community structure were primarily influenced by spatial distance limitations and vegetation type. Furthermore, we identified dominant fungal phyla with significant roles in energy cycling and organic matter degradation, including members of the *Sordariomycetes*, *Leotiomycetes*, *Archaeosporomycetes*, and *Agaricomycetes*. Functional analyses of soil fungal communities highlighted distinct microbial ecological functions in Xinjiang and Fujian soils. Xinjiang soil was characterized by a focus on wood and plant *saprotrophy*, and endophytes, whereas in Fujian soil the fungal community was mainly associated with ectomycorrhizal interactions, fungal parasitism, and wood *saprotrophy*.

**Discussion:**

Our findings suggest fungal communities in different climatic conditions adapt along distinct patterns with, plants to cope with environmental stress and contribute significantly to energy metabolism and material cycling within soil-plant systems. This study provides valuable insights into the ecological diversity of fungal communities driven by geological and environmental factors.

## 1 Introduction

The diversity of terrestrial ecosystems is correlated with the diversity of organismal adaptations (Lu et al., 2020), with broadleaf forests, coniferous forests, and grassland three of the main vegetation types found in most ecosystems (Geng et al., 2019). These differing vegetation types present significantly varied litter and understory environments. For example, soil organic carbon and soil nutrients in coniferous plantations are significantly lower than those in broadleaf plantations in subtropical China, while soil environmental factors in grasslands are also significantly different from those in coniferous forests (Wang et al., 2010; Zhang et al., 2021). Litter differences between coniferous forests, broadleaf forests and grasslands have been show to reflect differences in microbial diversity (Rime et al., 2015). Many studies have focused on aboveground plant communities in terrestrial ecosystems, examining plant diversity (Isbell et al., 2011), spatial organization and structure of plant communities (Nakagawa et al., 2013), and ecological service functions (Peng et al., 2023). Aboveground plant communities and underground microorganisms are interrelated and interact with each other (Singh and Gupta, 2018). Although soil microorganisms play an important role in the stability and function of terrestrial ecosystems (Singavarapu et al., 2023), our understanding of the relationship between plants and soil microorganisms, particularly fungi remains limited. In addition, the spatial distribution characteristics of soil fungi in terrestrial ecosystems, comparing different climatic regions, has been poorly examined.

Fungi are components of terrestrial biodiversity and play important roles in ecosystem processes including energy cycling, remediation and in-/organic matter turnover, nutrient (e.g., carbon, nitrogen, phosphorus, and water) availability, and soil and mineral formation (Averill et al., 2014; Rusterholz et al., 2023). They play a role in plant and animal growth, development, and parasitism, helping to maintain the stability of ecosystem functions (Chen et al., 2020). Soil fungi, both free living and ecto-/endophytic can affect plant roots through the underground food chain, including altering the physical and chemical properties of the soil (Ding et al., 2019). In turn, plants can affect the surrounding microbial communities through the microenvironment of their roots (Berg and Smalla, 2009). Corresponding soil fungi can also affect aboveground plant communities by changing soil nutrient composition, by altering physicochemical properties of soils, and/or by regulating plant coexistence (van der Heijden et al., 2008). Studying the relationship between aboveground plants and underground soil fungal diversity can help in the development of strategies to improve conservation, maintenance of the stability of terrestrial ecosystems, and stress resistance. Previous studies have shown that soil fungi in different habitats have significantly different growth characteristics and transmission capabilities (Han W. et al., 2021), and fungi are usually highly sensitive to environmental changes (He et al., 2022). However, little is known concerning the extent to which spatial distribution characteristics of soil fungal communities are consistent in ecosystems of the same vegetation type but in significantly different climatic regions.

Soil fungal and vegetation species composition of different habitats depends on the spatial distribution of environmental requirements and conditions, resulting in unique biodiversity patterns (Legendre et al., 2009). Although it is well known that different climatic environments with different vegetation types have different associated microbiomes (Han X. et al., 2021; Fu et al., 2022), far less is known concerning correlations to fungal diversity. Fujian and Xinjiang represent two very different climatic regions, with consequent geographical environments deferring, however, they share some similarity in vegetation type (broad leaved forests), and thus provides a unique opportunity for comparison of soil fungal diversity. Fujian is located in the subtropical monsoon climate zone (coastal Southeast China), has a warm and humid climate, and is rich in vegetation types that include evergreen broadleaf forests and temperate coniferous forests. Xinjiang is in an arid continental climate zone (Western plateau China), where the climate is dry and cold, and the vegetation mainly includes grassland, deciduous broadleaf forests and evergreen coniferous forests. Albeit significantly different, both regions contain broadleaf and coniferous forests, thus providing a unique comparative context to study the structure and diversity of soil fungal communities.

We hypothesized that: (1) differences in microbial diversity and community composition between similar forest types in the two climate zones would correlate with soil physiological parameters, and (2) the relationship between soil microbial communities and surface plant communities would vary between the same vegetation types in the two climatic zones. To address these hypotheses, we used high-throughput sequencing to characterize the microbial community of soil samples in Fujian and Xinjiang. We defined the types of vegetation habitats according to the similarity/differences of vegetation conditions and analyzed the relationship between soil fungal community structure and diversity, plants and abiotic factors in the defined microbial habitats.

## 2 Materials and methods

### 2.1 Site description

**FIGURE 1:**
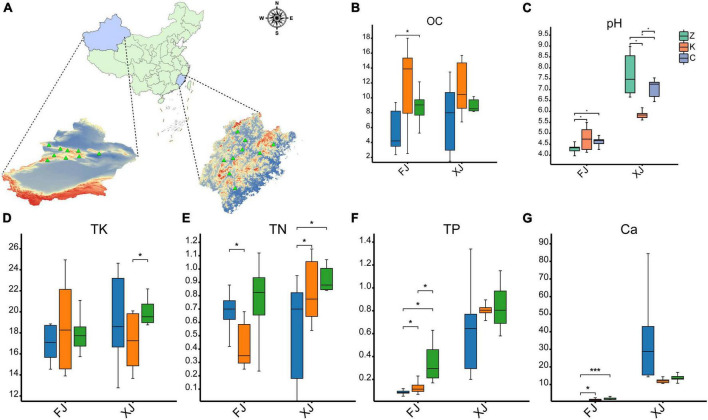
Comparison of physical and chemical properties of soil samples. **(A)** Map of location of isolation of soil samples from different provinces/regions in China (*n* = 36). **(B–F)** Soil physical and chemical properties as follows: **(B)** soil organic matter, **(C)** pH, **(D)** total potassium (TK), **(E)** total nitrogen (TN), **(F)** total phosphorus (TP). **(G)** the metal ion Ca2^+^ in the soil. The symbol * indicates *p* < 0.05, while the symbol *** indicates *p* < 0.001. FJ: soil samples from Fujian province, XJ: soil samples from Xinjiang Autonomous Region.

This study was conducted in Fujian Province (115° 50′ E-120° 43′ E, 23° 31′ N-28° 18′ N) and Xinjiang Autonomous Region (73° 40′ E-96° 23′ E, 34° 22′ N-49° 10′ N, Figure 1). The study area Fujian belongs to one of the five major climate types found in China (National Meteorological Administration [NMA], 1979). The study area of the Xinjiang Autonomous Region belongs to the temperate continental climate type, and the linear distance between the two is 4,283 km. The annual average temperature of Fujian Province in 2021 was 20.8°C, and the annual average temperature of each county/city was between 16.3 and 23.3°C, increasing from north to south. The average precipitation of the province was 1477.1 mm, and the maximum annual precipitation was 1860.7 mm, and the minimum was 1058.5 mm. In 2021, the average temperature in Xinjiang was 8.9°C, with extreme weather patterns that included four defined blizzards, three extreme rainstorms, seven cold waves and two low temperature events within the year, and an average precipitation of 162.2 mm (data from China Meteorological Data Service Center).

### 2.2 Experimental design and sampling

In July 2021, according to the different vegetation types in the study area, evergreen coniferous forests (XZ), deciduous broadleaf forests (XK), grasslands (XC) in Xinjiang and temperate coniferous forests (FZ), evergreen broadleaf forests (FK) and grasslands (FC) in Fujian were selected as sampling areas. Six 10 × 10 m repeated standard plots were established in each vegetation type plot. Each plot followed a five-point sampling method in which five soil samples were collected from the 0–15 cm soil layer near the plant roots. Soil samples from the same grid were thoroughly mixed into a sterile bag. A total of 36 soil samples were collected and divided into two parts, one of which was stored in a refrigerator at −80°C. The other portion of the soil sample was air-dried to constant weight at 30°C. The roots, stones and other debris in the soil samples were removed, and then the soil samples were sieved by 5 and 2 mm for soil physical and chemical properties analysis (Qi et al., 2018). Soil pH value was measured by a glass electrode pH meter, total nitrogen content was determined through the potassium dichromate-sulfuric acid digestion method, total phosphorus was determined using the sulfuric acid-perchloric acid digestion method, total potassium was determined by using a NaOH melting-flame photometer. Ca^2+^ and Mg^2+^ were determined through complex ometric titration, and Mn^2+^ was determined using atomic absorption spectrophotometer. Total phosphorus and total potassium contents were determined as described by Hengl et al. (2021).

### 2.3 DNA Extraction, polymerase chain reaction

Fungal DNA was extracted from 1 g of soil samples using the Fast DNA Spin Kit for Soil kit method. PCR amplification was performed using ITS1 (5′-CTTGGTCATTTAGAGGAAGTAA-3′) and ITS2 (5′-GCTGCGTTCTTCATCGATGC-3′) as fungal universal primers (Zhu et al., 2019). The PCR reaction system included in a 50 μL reaction mixture was: 25 μL 2 × San Taq PCR Mix, 2 μL upstream and downstream primers (10 μmoL/L), 2 μL DNA template, and ddH_2_O to 50 μL. The PCR amplification procedure was as follows: pre-denaturation at 95°C for 3 min, followed by 34 cycles, each cycle including 98°C for 1 min, 55°C for 30 s, 72°C for 30 s, and finally 72°C for 10 min.

### 2.4 Bioinformatic analysis

PCR amplification products were sequenced using the PacBio sequencing platform (Beijing Baimaike Biotechnology Co., Ltd). The resultant ITS gene sequences were processed by Lima v1.7.0 software, with barcode CCS identified to obtain the original Raw-CCS sequence data. Primer sequences were identified and removed by Cutadapt 1.9.1 software and filtered to obtain clean CCS sequences without primers. Next, UCHIME v4.2 software was used to identify and remove any chimera sequences to obtain an Effective-CCS sequence, and reads were clustered to obtain OTUs at a similarity level of 97.0% using the Usearch software. Taxonomic annotations were assigned to the feature sequences utilizing the naive Bayes classifier in conjunction with sequence alignment against the UNITE reference database, providing species classification information for each feature. Subsequently, community composition at various taxonomic levels (phylum, class, order, family, genus, species) were determined. Species abundance tables at different taxonomic levels were generated using the QIIME software.

Unless otherwise specified, all data were processed using the R programming language. The significance level for all tests was set at *p* < 0.05. We conducted data normality tests using independent sample *t*-tests. For non-normally distributed data, we applied log10, square root, or sine transformations to achieve a normal distribution. Parametric or non-parametric tests were used for normally or non-normally distributed data, respectively. The correlation analysis between soil physicochemical data in Fujian and Xinjiang was performed using independent sample *t*-tests for normally distributed data or Wilcoxon tests for non-normally distributed data.

Canonical Correspondence Analysis (CCA) was conducted using the vegan package in RStudio to explore the relationship between community structure and environmental factors. In addition, we used the vegan package in RStudio to visualize the similarity and dissimilarity of fungal community structures between the two regions through Non-Metric Multidimensional Scaling (NMDS) plots and Principal Coordinates Analysis (PCoA). Heatmaps depicting the relationship between species and environmental factors were generated using the pheatmap package in RStudio. FUNGuild was employed for predicting the functional profiles of fungal communities. Finally, CCA in R software was employed to reveal the physicochemical parameters that best explained variations in microbial community composition.

To assess the relative importance of stochasticity and determinism in the assembly of fungal communities in two regions, we evaluated the goodness of fit of the Sloan neutral community model (Sloan et al., 2006; Rosindell et al., 2011). The output plots of the neutral community model (NCM) primarily display the goodness of fit of the neutral model (R^2^) and the migration rate (m), as well as the predictions of the neutral model and their corresponding 95% confidence intervals (lines).

## 3 Results

### 3.1 Analysis of physicochemical properties

Comparing the soil samples derived from Fujian and Xinjiang revealed that the pH of the soil in the two climatic zones was significantly different (*P* < 0.01). The soil pH of Fujian averaged 4.56 ± 0.41, while the soil pH of Xinjiang averaged 6.87 ± 1.00. There were no significant differences in soil pH values among the three different vegetation types in the Fujian province. However, a significant difference in soil pH was observed between the deciduous broadleaf forests and evergreen coniferous forests and grasslands in Xinjiang (*P* < 0.01). In contrast, no significant difference in soil pH was found between the evergreen coniferous forest and grassland in the Fujian province. There were also no significant differences in soil organic matter between the sampled habitats of the two provinces (*P* > 0.05), but the soil organic matter content in the broadleaf forests in Fujian and Xinjiang was higher than for evergreen coniferous forests and grasslands. The phosphorus (P) content in Xinjiang soil samples was significantly higher than that in Fujian soil samples (*P* < 0.01). There was no significant difference in the P content of soil samples in the different regions within Xinjiang, but there was a significant difference in the P content between the soils in three habitats in Fujian, with the P content in grassland soil samples the highest (*P* < 0.05). There was no significant difference in total nitrogen (TN) and total potassium (TK) between the two regions.

**FIGURE 2:**
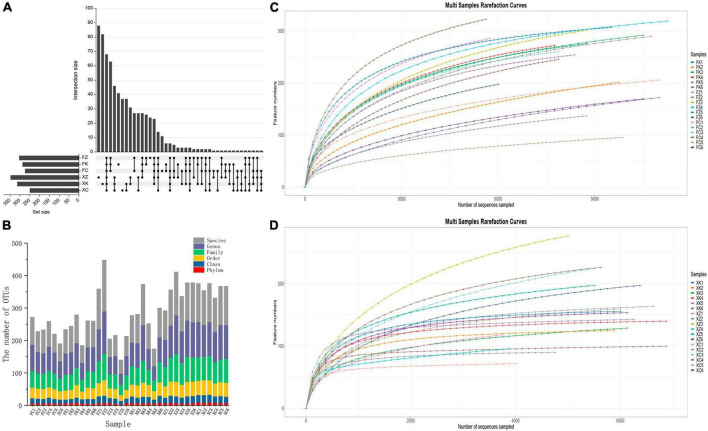
Operation taxonomic units (OTU) status identification and cluster analysis. **(A)** OTU (UpSet) analyses of soil samples from the different Fujian and Xinjiang regions. **(B)** OTU division and classification status identification. *X*-axis = sample ID, *Y*-axis = number of OTUs at each classification level (Phylum, Class, Order, Family, Genus, and Species. **(C)** Fujian and **(D)** Xinjiang dilution curves: *X*-axis = randomly selected ordinal number, *Y*-axis = corresponding number of species, with curves of different colors representing different samples.

A total of 196,419 high-quality sequences were obtained from the 18 soil samples obtained in the Fujian province by high-throughput sequencing after filtration, splicing and chimera removal. The sequence length ranged from 569 to 629 bp. A total of 754 fungal OTUs were detected, involving 11 phyla, 30 classes, 61 orders, 111 families and 192 genera. A total of 118,550 high-throughput sequences were obtained from the 18 Xinjiang derived soil samples after quality control, with sequence lengths ranging from 550 to 657 bp. A total of 917 fungal OTUs were detected, that were dispersed within10 phyla, 29 classes, 70 orders, 134 families and 266 genera. Differences in OTU levels in these samples are shown in the Upset plot (Figure 2A). The number of OTUs unique to deciduous broadleaf forests, evergreen coniferous forests, and grasslands in Xinjiang was 88, 88, and 37, respectively. The number of unique OTUs of soil fungi in the three different vegetation regions samples in Fujian was lower than that seen for the in Xinjiang samples. For Fujian province samples, there were 41 unique OTUs in the evergreen broadleaf forest, 24 unique OTUs in the coniferous forest and 11 unique OTUs in the grassland sampled regions. The number of fungal OTUs shared by the three different vegetation regions of Fujian and Xinjiang was only 82, accounting for only 10.9% of the number of OTUs in Fujian and 8.9% of the total number of OTUs in Xinjiang. OTUs distributions for each sample at different classification levels including phylum, class, order, family, genus, and species were determined (Figure 2B). The number of OTUs in samples at each classification level was relatively uniform and showed no significant increase. The variation trend of species richness with sequencing depth was sparse (Figures 2C, D). When the sequencing depth increased to 4000, all fungal curves for the Xinjiang soil samples reached a plateau, and similarly for the Fujian soil samples as the sequencing depth increased to 6000 for the fungal curves. The dilution curve of Fujian soil fungal samples was more deformed than that of Xinjiang soil samples, indicating that the community diversity of Fujian soil fungi was higher than that of Xinjiang soil fungi. As the dataset was increased in size, only a small number of low abundance species were detected, indicating that the sequencing depth gave good coverage of the species in the sample.

### 3.2 Alpha diversity analysis of fungal communities

The species richness and diversity indices of the 36 soil samples were determined at 97% similarity level (Table 1). α-Analyses of the soil fungal communities of Xinjiang and Fujian samples revealed similar trends in ACE and Chao1 indices for the Fujian and Xinjiang samples, with Fujian samples significantly higher than those sampled from Xinjiang. The Simpson index and Shannon index were used to estimate the species diversity of the six samples locations (3 each in Fujian and Xinjiang). These results showed that soil fungal species abundance in the Fujian sampled locations were significantly higher than that in Xinjiang, but the diversity of soil fungi in Fujian was not significantly different from that in Xinjiang.

**TABLE 1:** α-diversity analysis of different soil type samples.

Soil types	ACE	Chao1	Simpson	Shannon
Coniferous forests in Xinjiang Province (XZ)	200.62 ± 110.08bc	176.29 ± 100.38cd	0.91 ± 0.09ab	4.96 ± 0.68a
Broadleaf Forests in Xinjiang Autonomous Region (XK)	141.4 ± 19.2c	141.48 ± 19.09d	0.95 ± 0.02a	5.33 ± 0.54a
Grasslands of Xinjiang autonomous Region (XC)	216.5 ± 54.49b	217.63 ± 50.6bc	0.88 ± 0.09abc	4.62 ± 0.87a
Coniferous forests in Fujian Province(FZ)	348.44 ± 48.55a	327.79 ± 36.23a	0.9 ± 0.05ab	4.85 ± 0.85a
Broadleaf forests in Fujian Province (FK)	265.05 ± 43.16b	254.96 ± 36.07b	0.79 ± 0.11c	3.3 ± 0.63b
Grasslands in Fujian Province (FC)	336.82 ± 24.01a	331.33 ± 26.14a	0.84 ± 0.09bc	4.53 ± 0.68a

ACE, abundance-based Coverage Estimator represents the richness index used to assess the diversity of species in the samples; Chao1 stands for the Chao1 index in richness estimation, used to estimate the number of unobserved species and is one of the diversity metrics; Simpson, signifies the Simpson index, which measures the dominance of species in species diversity; higher values indicate lower diversity; Shannon denotes the Shannon diversity index, used to measure both species diversity and evenness in the sample; higher values indicate higher diversity. Means with different superscript letters in the same column are significantly different (*P* < 0.05).

### 3.3 Comparative analysis of beta diversity

**FIGURE 3:**
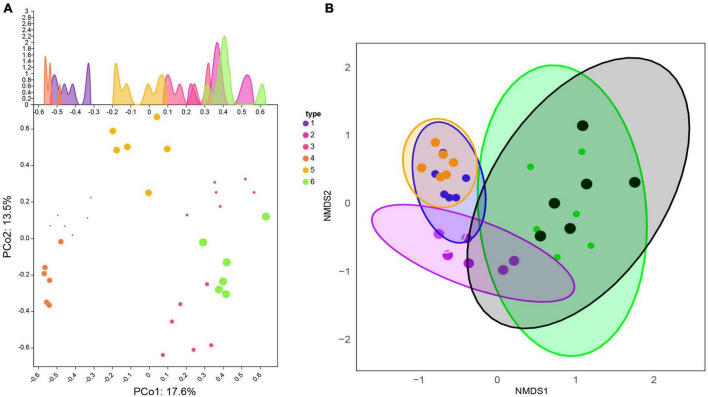
Comparison of β-diversity differences within and between Xinjiang and Fujian soil samples. **(A)** Principal Coordinate Analysis (PCoA). The horizontal and vertical coordinates represent two different principal components. **(B)** Similarity of fungal communities among different types of soils based on Non-metric Multidimensional Scaling (NMDS) analysis.

Principal Co-ordinates Analysis (PCoA) was used to examine the similarity/dissimilarity of sample community composition (Figure 3A). The results showed that the microbial community structure in the same sampling area was similar. Principal component 1 (PCoA1) and principal component 2 (PCoA2) explained 31.1% of the total variation and could be identified as the main source of variation. The samples along the first axis were clearly separated, indicating that the largest variation between samples come from soil samples from different habitats in the two regions (PCoA1 contribution rate was 17.6%). The analysis of the second principal component (PCoA2) showed that it explained 13.5% of the soil heterogeneity, likely due to variation in the number of sample repeats within the group.

Similar to PCoA, non-metric multidimensional scaling analysis (NMDS) was also applied to reveal the effects of soil at different sampling locations on fungal community composition (Figure 3B). These analyses indicated significant differences in fungal communities between coniferous forests, broadleaf forests, and grasslands between the two climate zones of Xinjiang and Fujian. The distribution of the 36 samples was relatively scattered, but samples from the same area clustered, indicating that fungal community composition was significantly different between soils from different regions and different habitats in the same region.

### 3.4 Analysis of taxonomic composition of dominant fungal populations between samples

**FIGURE 4:**
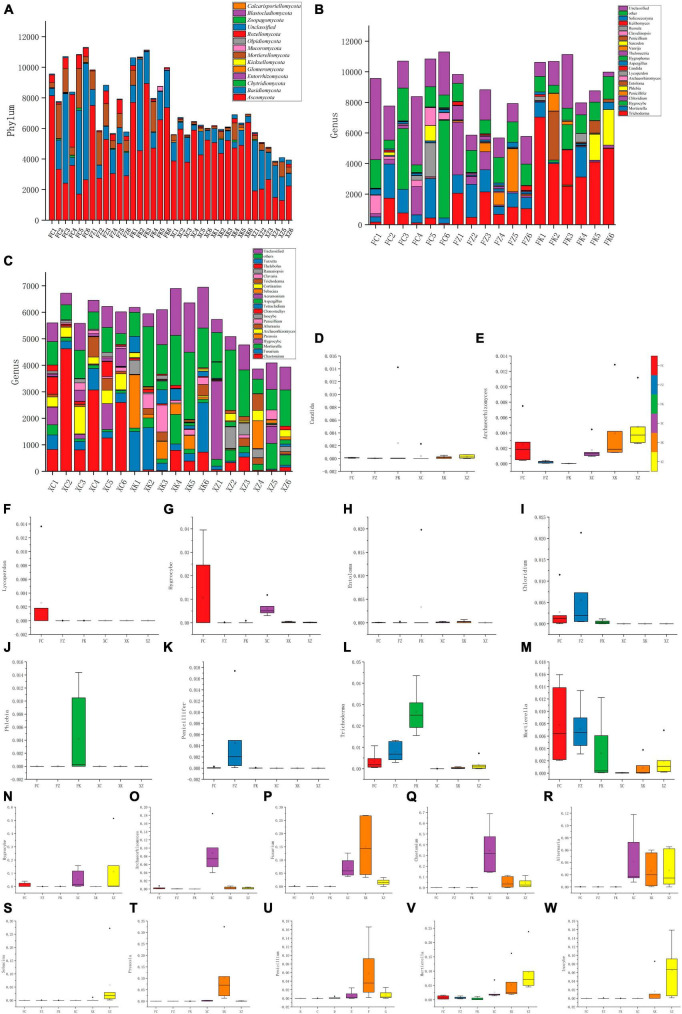
Composition of fungal communities at different taxonomic levels in all samples. **(A)** Relative abundance distribution of dominant fungal phyla. *X*-axis = sample ID, *Y*-axis = relative abundance of dominant fungal phyla. **(B,C)** The relative abundance of the same phylum at a deeper taxonomic level (genus). **(D–M)** Distribution of the top 10 fungal genera with the highest abundance in Fujian and Xinjiang soils. **(N–W)** Distribution of the top 10 fungal genera with the highest abundance in Xinjiang and Fujian soil. *X*-axis = the six samples, *Y*-axis = relative abundance of the genera in the corresponding samples.

Analysis of the sequencing results indicated that within the Fujian samples a total of 11 phyla and 192 genera of fungi were identified, with three phyla (the Entorrhizomycota, Kickxellomycota, and Zoopagomycota) being unique to Fujian. In Xinjiang, 10 phyla with 267 genera of fungi were identified, with two phyla (the Blastocladiomycota and Calcarisporiellomycota) uniquely to the Xinjiang soil samples. Significant differences in the dominant fungal phyla between the soil samples from the two regions were seen, with Fujian soil samples having higher species abundance at the phylum level compared to Xinjiang samples (Figure 4A). The top four dominant fungal phyla in both regions were the same (in order of proportion): Ascomycota, Basidiomycota, Mortierellomycota, and Rozellomycota. The Ascomycota dominated in Xinjiang grasslands, deciduous broadleaf forests, and in Fujian evergreen broadleaf forests and temperate coniferous forests. However, in Fujian grasslands and Xinjiang evergreen coniferous forests, there was no significant difference between the Ascomycota and Basidiomycota, with the Basidiomycota more prevalent than the Ascomycota in the Xinjiang evergreen coniferous forest soil samples examined.

At the genus level, the relative abundance analyses revealed that *Trichoderma*, *Mortierella*, and *Hygrocybe* were the dominant genera in Fujian grasslands (Figures 4B, C). *Chloridium*, *Trichoderma*, and *Mortierella* were the most dominant genera in Fujian coniferous forests, whereas *Phlebia*, *Entoloma*, *Trichoderma*, and *Mortierella* were found to be abundant in Fujian broadleaf forests. *Chaetomium*, *Fusarium*, and *Archaeorhizomyces* were the dominant genera found in Xinjiang grassland soil samples, and the dominant genera in Xinjiang deciduous broadleaf forests were *Preussia*, *Chaetomium*, *Fusarium*, and *Penicillium*, while the dominant genera in Xinjiang evergreen coniferous forests were different from those found in broadleaf forests and more similar to those identified in Fujian grasslands, with *Inocybe*, *Hygrocybe*, and *Mortierella* being dominant.

A comparison between the abundance differences between multiple samples and the compositional differences at each taxonomic level was performed with statistical tests. By selecting the top 10 genera with the highest abundance in Xinjiang and Fujian samples, a total of 20 genera were analyzed (Figures 4D–W). These data revealed that *Inocybe* was significantly distributed in Xinjiang coniferous forests, while the abundance of *Hygrocybe* in Fujian grasslands was significantly higher than in the other five soil samples. Additionally, these analyses indicated that *Mortierella*, unlike other genera, was widely present in soil samples from both regions.

### 3.5 Correlations between dominant fungal communities and soil factors

**FIGURE 5:**
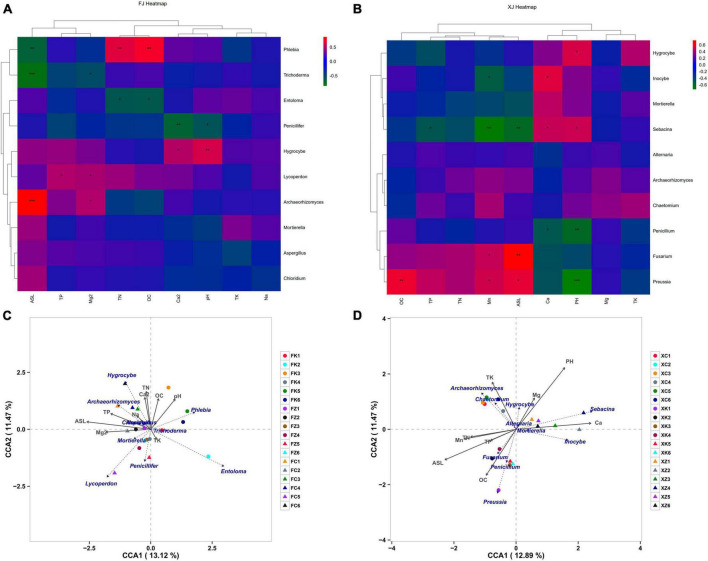
Correlation analysis of environmental factors and dominant species in soil samples, **(A,B)** represent the correlation analysis of soil microbial community composition and soil physicochemical factors based on Spearman correlation in Fujian and Xinjiang, respectively. **(C,D)** Represent Canonical Correspondence Analysis (CCA) of fungal communities based on soil physicochemical properties in Fujian Province and Xinjiang Province, respectively. Note: The horizontal axis represents soil physicochemical factors, the vertical axis represents dominant fungal at the genus level in each sample. Colors represent Spearman correlation, and asterisks indicate significance (*P < 0.05; **P < 0.01; ***P < 0.001).

Spearman correlation analyses were employed to explore the relationship between soil physicochemical parameters and fungal community structure in two climatic regions (Figures 5A, B). The results indicated significant variations in fungal species composition due to different soil nutrient levels. Notably, the genus *Mortierella* exhibited no significant correlations with the measured factors in both climatic regions, suggesting potential environmental adaptability and tolerance of this genus. *Hygrocybe* showed consistent correlations with environmental factors, exhibiting significant positive correlations with pH in both regions. In addition to *Hygrocybe*, pH in Xinjiang soil also displayed a significant positive correlation with the genus *Sebacina* but a highly significant negative correlation with the dominant genera *Preussia* and *Penicillium* in Xinjiang deciduous broadleaf forest soil. *Archaeorhizomyces* in Fujian soil showed a highly significant positive correlation with elevation and a significant correlation with Mg^2+^, while in Xinjiang soil, *Archaeorhizomyces* exhibited no significant correlations with environmental factors. Furthermore, elevation in Xinjiang soil was significantly positively correlated with the dominant genera *Preussia* and *Fusarium* but significantly negatively correlated with *Sebacina*. It also displayed a highly significant negative correlation with the dominant genera *Phlebia* and *Trichoderma* in Fujian soil. In Xinjiang soil, with the exception of the correlation between TP and the genus *Sebacina*, total nitrogen (TN), total phosphorus (TP), and total potassium (TK) did not exhibit significant correlations with the dominant genera associated with three environmental factors, which differed from the microbiota in Fujian soil. In Fujian soil, TN exhibited a highly significant positive correlation with *Phlebia* and a significant correlation with *Entoloma*, while *Lycoperdon* showed a significant positive correlation with TP.

Based on differences in soil physicochemical properties and fungal composition at the genus level, non-biological environmental driving factors influencing fungal community composition were identified using RDA (Figures 5C, D). The explained variation in fungal diversity by environmental variables was 24.59% for Fujian soil and 24.36% for Xinjiang soil. The results revealed that in Fujian soil, *Hygrocybe* and *Archaeorhizomyces* were mainly driven by TP, TN, and Na^+^, while *Phlebia* was primarily driven by pH. In Xinjiang soil, TP, TN, ASL, and OC primarily drove the dominant genera *Fusarium*, *Penicillium*, and *Preussia* in Xinjiang deciduous broadleaf forest. *Chaetomium* and *Archaeorhizomyces* were the dominant genera in Xinjiang grassland, primarily driven by the non-biological factor TK, consistent with the results presented in Figure 5B.

### 3.6 Functional analysis of soil fungal communities

**FIGURE 6:**
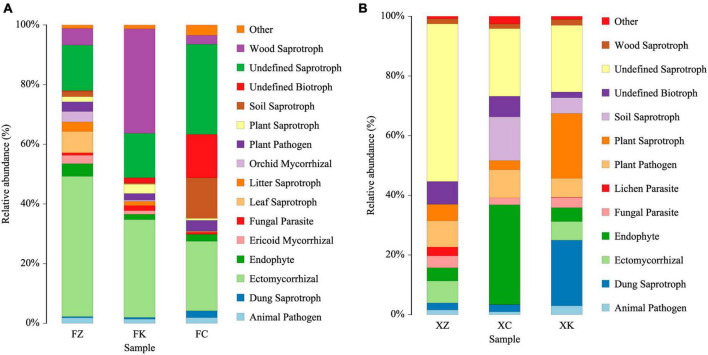
FUNGuild analyses predicting ecological functions of identified fungi. **(A)** Relative abundance of microbial community functions in Fujian soil samples. **(B)** Relative abundance of microbial community functions in Xinjiang soil samples.

Based on predictions using FUNGuild, the ecological functions of fungi were inferred (Figure 6). These analyses indicated that the fungal community in Xinjiang soil was primarily involved in wood saprotrophy, soil saprotrophy, plant nutrient provision, and facilitation of other material exchanges in the soil. In contrast, the predicted dominant ecological functions of fungal microorganisms in Fujian soil were focused on wood saprotrophy and ectomycorrhizal associations. Moreover, a significant portion (20–50%, *P* < 0.01) of the fungal community in Xinjiang soil samples was categorized as saprotrophs with undefined ecological functions.

### 3.7 Neutral community model analysis

**FIGURE 7:**
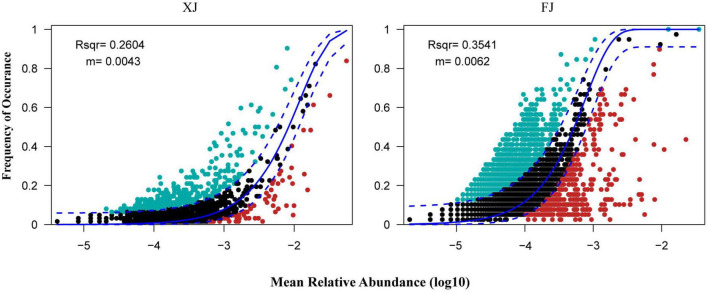
Fit of the neutral community model (NCM) in two regions. The solid blue lines indicate the best fit to the NCM as in Sloan et al. (2006), and the dashed blue lines represent the 95% confidence intervals around the model prediction. The OTUs that occur more or less frequently than predicted by the NCM are shown in aquamarine or red colors, respectively. R^2^ indicates the fit to the NCM. The m represents the migration rate at the community level.

Assembly mechanisms of the fungal communities in different regions and three different vegetation types using the neutral community model (NCM). Based on the OTUs dataset, the NCM explained a relatively low proportion of microbial assembly (The R^2^ values for Xinjiang and Fujian were 0.260 and 0.354, respectively, and the migration rates were 0.004 and 0.006, respectively) (Figure 7). These results suggest that the assembly of fungal communities in the two regions is primarily influenced by deterministic processes.

## 4 Discussion

### 4.1 Effects of environmental factors on microbial community structure

Soil ecosystems mediate material (e.g., carbon, nitrogen, and phosphorus) exchange, energy transfer, and act as the conduit for information communication among various animals, plants, and microorganisms (Massalha et al., 2017; Kumawat et al., 2022). As crucial members of the carbon and nitrogen cycles, microorganisms can respond rapidly to environmental changes, help remediate inorganic and organic matter, and form intimate associations in networks with other microbes, plants, and animals (Falkowski et al., 2008; Ma et al., 2023). Previous studies have indicated that the structure and diversity of soil microbial communities are influenced by environmental factors including pH, nitrogen, phosphorus, potassium, temperature, humidity, and other climatic conditions (Xu et al., 2014; Aßhauer et al., 2015) as well as related to geographic parameters (Hanson et al., 2012), however most such studies have focused on bacterial populations and not fungal. As expected, our data indicate significant differences in soil pH value, nitrogen, phosphorus, potassium, and organic matter content between the coastal regions of Fujian and the remote inland areas of Xinjiang. Fujian soils were acidic, with pH values ranging from 3.97 to 5.5, whereas the soil locations sampled in Xinjiang were predominantly alkaline, with only the Xinjiang broadleaf forest exhibiting acidic pH value (5.61), with the rest ranging above that to a pH of 8.98. A negative correlation was seen between soil pH and soil fungal abundance and diversity, which differed from a previous study (Rousk et al., 2009), but may be more broadly consistent with most fungi preferring acidic conditions. In addition, many fungi acidify their environment thus contribution to acidification of soils. Thus, it is possible that the alkaline soil in Xinjiang is less hospitable to fungi and/or since there are less fungi in the Xinjiang soils, less (fungal) mediated acidification occurs, and hence the soil is more alkaline.

Phosphorus content in the root zone soils of the three vegetation types sampled in Xinjiang was significantly higher than in the (three different vegetation types) soils examined from Fujian. Nitrogen and potassium levels did not show the same trend. Organic matter content in broadleaf forests from both regions were similar (and highest of the soils tested). However, despite the higher organic matter content in the soils of broadleaf forests (of both Xinjiang and Fujian), this did not correlate with fungal diversity. This suggests that the fungi in the root zone soils of broadleaf forests may remain limited, consistent with what has been reported by others (Averill et al., 2014).

### 4.2 Alpha and beta diversity analysis of microbial community

To better examine total species abundance and microbial diversity, we selected a sample size validated by the species accumulation curve trend. Using α-diversity indices (Shannon, Simpson, Chao1, ACE) and examining the distribution of microbial communities at various taxonomic levels, we assessed fungal diversity and abundance. The Chao1 index showed that species richness and the number of unassigned species were significantly higher in the Fujian samples as compared to the Xinjiang soil samples. Overall, the Fujian soil samples exhibited higher fungal richness and diversity at the phyla and unassigned levels, while the Xinjiang soil samples were more diverse at the identifiable genus level.

Principal Component Analysis (PCA) and Non-metric Multidimensional Scaling (NMDS) were used to analyze the structural composition and variation of fungal soil community (Quast et al., 2013; Zhu et al., 2022). Although the distribution of the 36 samples (in the two-dimensional analytic space) was relatively dispersed, a clear clustering of samples from the same region (and even more so from the same location within the region) was seen. It is challenging to determine whether the observed differences in the fungal soil microbial community structure result from distinct geographical locations and varying vegetation root and cover communities, or if different fungal communities play a role in shaping the vegetation. The most likely scenario is that they mutually influence each other. However, there was also a certain degree of random variation among soil samples within the same region, which indicates considerable environmental plasticity of the soil fungi within the same “habitat.”

### 4.3 Analysis of dominant flora and functional flora

Our data reveal the presence of fungi from at least 31 distinct phyla in the soils of the Xinjiang and Fujian ecosystems. At the phylum level, the fungal soil root-associated microbiota in Fujian and Xinjiang province included 31 and 30 different class, respectively, of which 24 were shared (found in both). Five classes, the Archaeosporomycetes, Blastocladiomycetes, Calcarisporiellomycetes, Cystobasidiomycetes, and Paraglomeromycetes, were found to be unique to Xinjiang, with five other classes, the Entorrhizomycetes, Rhizophydiomycetes, Xylonomycetes, Zoopagomycetes, and Endogonomycetes, unique to Fujian. Eight classes, namely the Sordariomycetes, Agaricomycetes, Mortierellomycetes, Saccharomycetes, Eurotiomycetes, Tremellomycetes, Pezizomycetes, and Dothideomycetes, were highly abundant and shared between the two regions. Previous studies have reported the wide distribution of the Agaricomycetes in soil environments, where they play an important role in decomposition and transformation of soil organic matter, facilitating the breakdown and conversion of organic compounds (Hibbett and Donoghue, 2001; Moore et al., 2015; Yi et al., 2019).

Furthermore, Xinjiang soils had a relatively high abundance of the class Archaeosporomycetes, second only to the Sordariomycetes and Agaricomycetes in grassland soils. Archaeosporomycete spores and mycelia can interact with soil microorganisms, plant roots, and other fungi, establishing a mycorrhizal symbiosis with plant roots and providing water and nutrients to plants (Fan et al., 2022). This might be related to the arid and low-rainfall climate in Xinjiang. Sordariomycetes and Agaricomycetes, as functional fungal groups involved in soil organic matter, were found to be widespread across six habitats, consistent with previous research results. Due to their broad organic matter degradation capabilities, the Sordariomycetes and Agaricomycetes become the dominant fungi in plant root soils.

The fungal microbial community structure in Fujian soil was found to be dominated by the following top 10 genera—*Archaeorhizomyces*, *Candida*, *Chloridium*, and *Entoloma*. In Xinjiang coniferous forest soil, the dominant fungal genera included *Chaetomium*, *Fusarium*, *Mortierella*, *Inocybe*, and *Hygrocybe*. In addition, *Trichoderma*, known for various plant beneficial effects, including protection (of plants) from pathogenic microorganisms (Sharma et al., 2017) and disease (Woo et al., 2023), was found to be a dominant fungal genus in Fujian. On the other hand, certain types of *Fusaria*, a dominant fungal genus identified in the Xinjiang soil samples, are plant pathogens (Berasategui et al., 2023). However, some *Fusaria* may have beneficial functions, such as organic matter decomposition and nutrient cycling (Mukjang et al., 2022). Furthermore, one of the dominant genera (*Sebacina*) found in Xinjiang is known to form symbiotic relationships with plant roots, promoting plant growth by providing nutrients and enhancing plant tolerance to environmental stress (Weiss et al., 2016; Lupini et al., 2023). These fungal functional groups can help plants enhance their tolerances to environmental stress and play crucial roles in plant growth under long-term environmental stress conditions (Weiss et al., 2011; Perez-Lamarque et al., 2022). While not all dominant fungal species can be attributed to directly aiding plants in stress management, their distinct microbial characteristics, in some instances, are likely linked to alterations in the soil environment. Significant variations in genera distribution were observed across different samples, suggesting that differences in geological habitats, soil properties, and climatic conditions either contribute to these variations and/or could be, even if to a small degree, determined by their respective fungal communities. However, our findings suggest that fungal community structures are influenced by spatial distance, potentially due to limitations in dispersal and interactions with environmental heterogeneity (Fierer et al., 2005; Ferrari et al., 2016). Thus, environmental conditions and geographic variations play a crucial role in shaping these fungal microbial communities.

Our canonical correspondence analysis (CCA) results showed a significant positive correlation between the fungal genera *Hygrocybe* and *Archaeorhizomyces* in Fujian soil and nitrogen and phosphorus content, consistent with these fungal groups being involved in soil material cycling and energy flow processes. In Xinjiang, the abundance of *Fusarium* and *Penicillium* may indicate their participation in nitrogen and phosphorus energy cycling that could significantly alter the nutrient characteristics of the soil. Soil pH was positively correlated with many of the identified dominant fungal populations, confirming the predictive role of pH as a major abiotic driver in shaping soil fungal communities (Tan et al., 2020; Yang et al., 2020).

Herein, the ecological functions of soil fungi have been a focal point of interest. Comparative analysis, using tools such as FUNGuild (Nguyen et al., 2016), revealed that the dominant fungal microbial communities in Fujian soil mainly consisted of ectomycorrhizal, soil saprotrophs, and wood saprotrophs. In contrast, the dominant fungal microbial communities in Xinjiang soil were primarily composed of endophytes, plant saprotrophs, dung saprotrophs, and intriguingly a significant number of undefined saprotrophs. It is well-known that mycorrhizal fungi play a crucial role in enhancing plant nutrient uptake and supply, improving stress resistance, and influencing the structure and diversity of plant communities (Harrier and Watson, 2004). These fungi form mutualistic associations with specific plant species, leading to the dominance of certain plant species in soils with ectomycorrhizal symbiosis. The results of the community functional analysis performed as part of this work showed that mycorrhizal fungi in Xinjiang constituted only a small fraction of the total, while the majority was unidentified saprophytic fungus. However, mycorrhizal fungi, as beneficial symbionts of plant roots, form symbiotic relationships with over 70% of plant species worldwide (Gao et al., 2019). Therefore, we speculate that some of the saprophytic fungi present in the root soil microbiomes of the three vegetation types in Xinjiang may possess partial functions similar to mycorrhizal fungi. These data also highlight a significant reservoir of uncharacterized fungi, and further research is needed to explore the microbial communities in these soils in greater detail.

The NCM results indicated that the assembly processes of fungal communities in Fujian and Xinjiang are primarily influenced by deterministic processes. Deterministic processes may include environmental filtering and interspecies competition, which play important roles in both regions (Stegen et al., 2012). However, compared to Fujian, Xinjiang exhibits a greater influence of deterministic processes on fungal community assembly, potentially due to its unique environmental conditions and selective pressures. Furthermore, the higher migration rate observed in Xinjiang suggests that the fungal communities in this region have higher dispersal abilities and may have stronger connectivity with other fungal communities. This could be one of the reasons for the observed differences in community assembly processes between the two regions.

## 5 Conclusion

Climate variations and spatial distances collectively contribute to the evolution of soil heterogeneity, thereby influencing the structure and diversity of soil fungal communities. In this study, by comparing two distinct regions, the coastal Fujian and the far inland Xinjiang areas, with three distinct regions within each of these areas, we identified the main fungal taxa in these ecosystems. Fungi involved in energy cycling and organic matter degradation were identified, including *Sordariomycetes*, *Agaricomycetes*, *Mortierellomycetes*, *Saccharomycetes*, and others. In addition, ecological functional differences were observed among the dominant fungal microbial communities between soils with different vegetation root systems. For instance, most of the fungal communities in Xinjiang grassland soil were endophytic fungi, likely contributing to enhancing plant resistance to environmental stress, and hence selected for by the plants as well exerting selective pressure on specific plants. In contrast, Fujian grassland soil exhibited a significant presence of fungal plant pathogens and parasites that impact plant growth, potentially indicating that pathogenic processes are more active in these ecosystems. Our results provide important insights into the interactions among soil microorganisms in different vegetation types and the primary functional roles of fungal microbial communities under the constraint of spatial distances.

## Data availability statement

The original contributions presented in this study are included in this article/supplementary material, further inquiries can be directed to the corresponding authors.

## Author contributions

SL: Conceptualization, Methodology, Writing – original draft. CX: Data curation, Writing – original draft. LL: Data curation, Writing – original draft. NK: Writing – review and editing. MZ: Data curation, Writing – original draft. ZZ: Data curation, Writing – original draft. WZ: Resources, Writing – original draft. CY: Resources, Writing – original draft. HS: Writing – original draft. PL: Writing – review and editing. XG: Writing – review and editing. JQ: Conceptualization, Funding acquisition, Resources, Supervision, Writing – review and editing.
